# Bromodomain-containing protein 4 regulates interleukin-34 expression in mouse ovarian cancer cells

**DOI:** 10.1186/s41232-020-00129-4

**Published:** 2020-10-14

**Authors:** Nanumi Han, Delnur Anwar, Naoki Hama, Takuto Kobayashi, Hidefumi Suzuki, Hidehisa Takahashi, Haruka Wada, Ryo Otsuka, Muhammad Baghdadi, Ken-ichiro Seino

**Affiliations:** 1 Division of Immunobiology, Institute for Genetic Medicine, Hokkaido University, Kita-15 Nishi-7, Kita-ku, Sapporo, Hokkaido 060-0815 Japan; 2 Department of Molecular Biology, School of Medicine, Yokohama City University, 3-9 of Fukuura Kanazawa-ku, Yokohama, Kanagawa 236-0004 Japan

**Keywords:** Bromodomain-containing protein 4, Interleukin-34, Cytokine induction, Gene regulation, JQ1, Tumor cell biology, Tumor promotor

## Abstract

**Background:**

Interleukin (IL)-34 acts as an alternative ligand for the colony-stimulating factor-1 receptor and controls the biology of myeloid cells, including survival, proliferation, and differentiation. IL-34 has been reported to be expressed in cancer cells and to promote tumor progression and metastasis of certain cancers via the promotion of angiogenesis and immunosuppressive macrophage differentiation. We have shown in our previous reports that targeting IL-34 in chemo-resistant tumors in vitro resulted in a remarkable inhibition of tumor growth. Also, we reported poor prognosis in patients with IL-34-expressing tumor. Therefore, blocking of IL-34 is considered as a promising therapeutic strategy to suppress tumor progression. However, the molecular mechanisms that control IL-34 production are still largely unknown.

**Methods:**

IL-34 producing ovarian cancer cell line HM-1 was treated by bromodomain and extra terminal inhibitor JQ1. The mRNA and protein expression of IL-34 was evaluated after JQ1 treatment. Chromatin immunoprecipitation was performed to confirm the involvement of bromodomain-containing protein 4 (Brd4) in the regulation of the *Il34* gene. Anti-tumor effect of JQ1 was evaluated in mouse tumor model.

**Results:**

We identified Brd4 as one of the critical molecules that regulate *Il34* expression in cancer cells. Consistent with this, we found that JQ1 is capable of efficiently suppressing the recruitment of Brd4 to the promotor region of *Il34* gene. Additionally, JQ1 treatment of mice bearing IL-34-producing tumor inhibited the tumor growth along with decreasing *Il34* expression in the tumor.

**Conclusion:**

The results unveiled for the first time the responsible molecule Brd4 that regulates *Il34* expression in cancer cells and suggested its possibility as a treatment target.

## Background

Tumor microenvironment (TME) is composed of various types of cells including not only tumor cells but also immune cells, fibroblasts, mesenchymal, and endothelial cells. Cytokine is known as one of the important components for interactions and communications between tumor and non-tumor cells. Some cytokines promote tumor progression while others have anti-tumor effects. For example, interleukin (IL)-10 is involved in suppression of immune response via inhibiting the expression of major histocompatibility complex and costimulatory molecules on antigen-presenting cells which sometimes support tumor growth. On the contrary, IL-12 acts as an inducer of interferon-γ in anti-tumor immune responses [1–3]. Among various cytokines, IL-34 is a novel cytokine that was first identified in 2008 as an alternative ligand to colony-stimulating factor-1 (CSF-1) for the CSF-1 receptor (CSF-1R) [4]. IL-34 has been reported to play crucial roles in TME. To date, IL-34 expression was observed in various types of tumors such as lung, liver, or colon cancer [5–7]. It has been shown that IL-34 expression was upregulated in cancer cells upon stimulation with anticancer drugs that was implicated with cancer cells’ acquisition of resistance to chemotherapeutic treatment [8]. Additionally, although immune checkpoint inhibition therapy using anti-programmed death-1 antibody has shown dramatic effects, considerable cases with therapeutic resistance have been reported [9, 10]. Among those, IL-34 has been suggested as a driver molecule of the resistance by inducing immune suppressive macrophages [11]. It has been reported that the macrophages generated by stimulation through CSF-1R signaling have the potential to induce T cell exhaustion and dysfunction [12]. There is another clinical fact that IL-34 expression in cancer correlates with poor prognosis and higher disease stage in several types of cancers such as brain, lung, ovarian cancers, and melanoma [13]. Therefore, it seems important to understand the expression mechanism of IL-34 in cancer cells. According to a previous report, IL-34 production is regulated through NF-κB or c-Jun *N*-terminal kinase signaling pathway [14]. However, the transcriptional regulation of IL-34 has not been identified. Consequently, this study aims to clarify the transcriptional regulator that controls *Il34* expression.

There is a report indicating that the canonical promoter of *IL34*, as well as of *CSF1R*, is rich in putative RUNX1 binding sites in melanoma [15]. It has been noted that RUNX1 recruits the transcription regulators cyclin-dependent kinase (CDK), bromodomain-containing protein 4 (BRD4), the mediator complex, and the looping factor LIM domain binding 1 [16]. Among them, BRD4 is one of the components of bromodomain and extraterminal domain (BET) family with BRD2, BRD3, and BRDT. It has been reported that the BET family protein regulates cellular proliferation and cytokine production [17–19]. In detail, BRD4 has two different roles that plays as a transcriptional regulator and an epigenetic regulator (histone reader). As a transcriptional regulator, there is a report indicating that BRD4 interacts with RNA polymerase II (POL II) or P-TEFb complex (CDK9 and CycT1) [20]. As a histone reader, it was suggested that BRD4 recognizes histone lysine acetylation by recruiting additional chromatin modifiers [21, 22]. Because inhibition of BRD4 impedes growth of cancer cells, targeting BRD4 has recently emerged as a promising anticancer approach [20]. However, it has not been explored whether expression of IL-34 is regulated by these factors, such as BRD4.

JQ1, a low molecular compound which inhibits the binding of BET family members, including Brd4, to their targets, has been reported to modulate transcription of oncogenes such as c-Myc [23]. Moreover, JQ1 is known to inhibit multiple targets including *TNF-alpha*, *Il6*, and *Mcp1* both in vitro and in vivo [24]. Thus, JQ1 is now widely used in cancer research [25, 26], and its administration either alone or in combination with other anti-cancer agents has exhibited efficient suppression of a variety of tumors [27, 28].

In this brief report, we show for the first time that BET inhibitor JQ1 could reduce *Il34* expression in two IL-34 highly producing cancer cells, such as OV3121-ras4 and HM-1. Brd4 was recruited on *Il34* promotor region which was blocked by JQ1, leading to downregulation of IL-34 production. When JQ1 was administered into an in vivo tumor model, both *Il34* expression and tumor growth were suppressed. These results unveil the mechanism of IL-34 expression, which implicates a novel TME-targeting cancer therapy.

## Methods

### NCBI database of the original sample accessions list

The data of IL-34 transcript treated by inhibitors were collected from the NCBI database. The original sample series are listed below. Human melanoma cell line A375 treated by vemurafenib: GSE42872, samples were analyzed RMA normalized data processing by the Peter MacCallum Cancer Centre. Human MLL leukemia cell line RS4.11 treated by GSK-3: GSE19736, samples were analyzed with Expression Console (Affymetrix) data processing by Stanford University. Human breast cancer cell line MCF7 treated by bortezomib: GSE30931, samples were analyzed with log2 transformation of signal intensity, quantile normalization, R/Bioconductor, and BeadStudion data processing by the University Medical Center Göttingen. Human eosinophilic leukemia cell line EOL-1 treated by imatinib: GSE53633, samples were analyzed with MAS 5 algorithm in Expression Console (Affymetrix) data processing by the University of Bologna. Human ovarian cancer cell line SKOV-3 treated by SAHA: GSE53603, samples were analyzed with GC-RMA analysis data processing by Panagiotis Konstantinopoulos. MYC-amplified medulloblastoma cell line D458 treated by JQ1: GSE51020, samples were analyzed with GenePattern (The Broad Institute) using Affymetrix default analysis settings and RMA as normalization method by Dana-Farber Cancer Institute. Primary mouse malignant peripheral nerve sheath tumor tissue MPNST treated by PD-0325901 (PD-901): GSE57141, samples were analyzed with BRB array tools data processing by Harvard Medical School—Brigham Women’s Hospital.

### Cell culture and reagents

OV3121 and OV3121-Ras4 were obtained from professor Toshio Seyama (Department of Molecular Pathology, Research Institute for Radiation Biology and Medicine, Hiroshima University). Mouse ovarian cancer cell lines HM-1 were obtained from RIKEN BioResource Research Center (RIKEN BRC).

OV3121 cells were plated on culture dish pre-coated with 0.5 μg/mL laminin (Nippi, Tokyo, Japan) and cultured in RPMI 1640 medium (Fujifilm, Tokyo, Japan) supplemented with 10% fetal bovine serum (FBS) (Sigma-Aldrich Japan), 1% MEM non-essential amino acid (NEAA) (Nacalai Tesque, Kyoto, Japan), and 1% penicillin/streptomycin (P/S) (Nacalai Tesque). OV3121-Ras4 cells were cultured in Dulbecco’s Modified Eagle’s Medium (Fujifilm) supplemented with 10% FBS, 1% MEM NEAA, and 1% P/S. HM-1 cells were plated on culture dish pre-coated with 0.5 μg/mL laminin and cultured in Minimum Essential Medium-Alpha (Fujifilm) supplemented with 10% FBS, 1% MEM NEAA, and 1% P/S. Cells were maintained at 37 °C in incubator under an atmosphere containing 5% CO_2_.

JQ1 was purchased from Selleckchem (TX, USA) and diluted in dimethyl sulfoxide (DMSO) (Fujifilm). OV3121 and OV3121-RAS4 cells were treated with 10 μM of JQ1, and HM-1 cells were treated with 0.01 μM, 0.1 μM, 1 μM, or 10 μM (final concentration of DMSO 0.1% (vol/vol)) for 48 h. Control groups were treated with 0.1% (vol/vol) of DMSO.

### Generation of Il34-overexpressing cell lines

Mouse *Il34* cDNA was obtained by PCR-amplified from mouse brain cell’s cDNA. To obtain mouse *Il34* cDNA, we used primers as follows: forward 5′-ATGCCCTGGGGACTCGCCTG-3′ and reverse 5′-TCAGGGCAACGAGCCATGGC-3′. The obtained moue *Il34* cDNA was cloned into lentiviral vector, pLenti-EF1a-C-Myc-DDK-IRES-Puro (OriGene, MD, USA). Lenti-X293T cells (Takara bio, Shiga, Japan) were transfected with the lentiviral vector and two packaging plasmids pCMV-VSV-G-RSV-Rev (RIKEN BRC DNA BANK, Tsukuba, Japan) and pCAG-HIVgp (RIKEN BRC DNA BANK) using *TransI*T-X2 (Mirus Bio LLC., WI, USA), and incubated 3 days. After collection of supernatant, wild-type of HM-1 was cultured with 1:1 mixture of Lenti-X293T cell supernatant and fresh medium, following selection by puromycin.

### Enzyme-linked immunosorbent assay (ELISA)

The production of IL-34 in cell lines was measured with ELISA using LEGEND MAX^TM^ Mouse IL-34 ELISA kit with pre-coated plates (clone: Poly5193) (BioLegend, CA, USA). Culture supernatants were collected at 48 h after seeding the cells at a density of 2 × 10^5^ cells/well of 96-well plate. Absorbance at a test wavelength of 450 nm and a reference wavelength of 570 nm was measured by using a Multiskan FC (Thermo Fisher Scientific, MA, USA).

### Cell viability assay

Cell viability was evaluated using the MTT Cell count kit (Nacalai Tesque). Absorbance at a test wavelength of 570 nm and a reference wavelength of 670 nm was measured by using a Multiskan FC (Thermo Fisher Scientific, MA, USA). Cell proliferation was observed for up to 2 days.

### Quantitative reverse transcription PCR analysis

Total RNAs were extracted using TriPure Isogen Reagent (Roche Diagnostics, Mannheim, Germany), and 1 μg of total RNAs was used for first-strand cDNA synthesis using ReverTraAce (TOYOBO, Osaka, Japan). qRT-PCR was performed on cDNA products using Fast SYBR Green PCR Master Mix (Thermo Fisher Scientific), and samples were run on Applied Step One real-time PCR system (Thermo Fisher Scientific). The thermal cycling conditions were composed of 95 ^°^C for 20 s followed by an initial denaturation step at 95 ^°^C for 3 min, 40 cycles at 95 ^°^C for 3 s, and 60 ^°^C for 30 s. The experiments were carried out in triplicate. The relative quantification in gene expression was determined using the 2^-ΔΔCt^ method and normalized by *Gapdh*. Primers were as follows: *Gapdh* (forward: 5′-TCAAATGGGGTGAGGCCGGT-3′ and reverse: 5′-TTGCTGACAATCTTGAGTGA-3′) and *Il34* (forward: 5′-CTTTGGGAAACGAGAATTTGGAGA-3′ and reverse: 5′-GCAATCCTGTAGTTGATGGGGAAG-3′). All experiments were performed in triplicate for each sample.

### Chromatin Immunoprecipitation-qPCR (ChIP-qPCR)

Cells (5 × 10^6^) were cultured in a 10-cm dish and crosslinked with 2 mM disuccinimidyl glutarate crosslinker in PBS for 30 min and then 1% formaldehyde in PBS for 10 min at room temperature. The cells were harvested and lysed with 2 mL of ChIP lysis buffer. Cell lysates (1 mg/mL) were sonicated with a Bioruptor (Sonic Bio, Kanagawa, Japan) 15 times for 30 s each time. After sonication, Micrococcal Nuclease (Takara Bio, Tokyo, Japan) was added to digest DNA. Protein G Magnetic beads (Bio-Rad, CA, USA) and specific antibodies were added to the digested lysates and incubated for 2 h at 4 °C. Specific antibodies 10 μg of anti-Brd4 polyclonal antibody (Bethyl Laboratories, TX, USA), 5 μg of normal rabbit polyclonal IgG (Medical & Biological Laboratories, Japan), 2 μg of anti-H3K27ac polyclonal antibody (Abcam Japan), 2 μg of anti-H3K4me3 polyclonal antibody (Abcam Japan), and 30 μL of Dynabeads M-280 sheep anti-rabbit polyclonal IgG (Thermo Fishcer Scientific) were used in the assays. The beads were washed twice with ChIP buffer containing 200 mM KCl, 2 mM CaCl_2_, and 50 mM Tris-HCl pH 8.0, twice with 500 mM KCl ChIP wash buffer, and once with TE buffer. Bound complexes were eluted from the beads with 100 mM NaHCO_3_ and 1% SDS by incubating at 50 °C for 30 min. Cross-linking was reversed by overnight incubation at 65 °C. Immunoprecipitated DNA and input DNA were treated with RNase A and proteinase K by incubation at 45 °C. DNA was purified using the QIAquick PCR purification kit (Qiagen, MD, USA) or MinElute PCR purification kit (Qiagen). Immunoprecipitates and input were analyzed by quantitative PCR. To determine the Brd4 binding region on *Il34* gene, we chose four regions as follows: an estimated promoter region (R2), its upstream region (R1), and the gene region (R3 and R4). Primers were as follows: R1 (forward: 5′-GTGGTGGCACAAGCCTATAA-3′ and reverse: 5′-GCTGGGACAACATCTCTTTCT-3′), R2 (forward: 5′-TGTCTCAGGCTTTGGTGTTAG-3′ and reverse: 5′-TGGTTTGTTTGTTTGGCTTGT-3′), R3 (forward: 5′-GTGCCTTGGAGTCCTTTCTT-3′ and reverse: 5′-GAAGGAGGAGAGAGAGACTGATTA-3′), and R4 (forward: 5′-TACTCCAGGTGACAAGTCCT-3′ and reverse: 5′-TAGCTGAATCAACCACCATCC-3′). *Il34* primers used in ChIP assay of H3K27ac and H3K4me3 were as follows: R2 (forward: 5′-GAGAAGACTGTAGGCTGAACAC-3′ and reverse: 5′-GTCGCCGGAAGCTTTGA-3′), R3 (forward: 5′-TGAGCTGCAATGGGACTG-3′ and reverse: 5′-GGCCACCAAGTCCAGAAA-3′), and R4 (forward: 5′-TACTCCAGGTGACAAGTCCT-3′ and reverse: 5′-TAGCTGAATCAACCACCATCC-3′).

The ChIP signal was normalized to input. Three biological replicates were performed for each experiment.

### In vivo tumor assay

All experimental animals were maintained in our specific animal facility according to institutional guidelines, with protocols which have been approved by the institutional animal care and use committee of Institute for Genetic Medicine of Hokkaido University. B6C3F1 (Japan SLC, Shizuoka, Japan) female mice (6–8 weeks old) were injected subcutaneously with *Il34*^WT^ HM-1 or *Il34*^OE^ HM-1 cell lines (1 × 10^6^ cells). After 7 days, mice were divided into two groups randomly. JQ1 was dissolved in DMSO. The two groups were treated with vehicle control or JQ1 (50 mg/kg 3 times a week) for 3 weeks. During treatment, tumor size was measured 3 times a week, and tumor volumes were calculated as length×width×height. Tumors were then removed from sacrificed mice, photographed, and analyzed by qPCR.

### Statistical analysis

Significance was determined by Student’s *t* test. *p* value was considered statistically significant when < 0.05.

## Results

### BET inhibitor JQ1 suppresses IL-34 expression in IL-34-producing cancer cell lines

In order to find possible candidates that could regulate IL-34 expression, we have at first taken a glance at transcriptional changes of *IL34* in human cancer cell lines and a tumor tissue treated with several low molecular inhibitors. Data were gathered from GEO profile of NCBI database [29], and we found that among those only BET inhibitor JQ1 showed downregulation of *IL34* expression (reference series: GSE51020) (Supplementary figure 1). Thus, we attempted to analyze *IL34* expression which seemed to be regulated by JQ1.

**Fig. 1:**
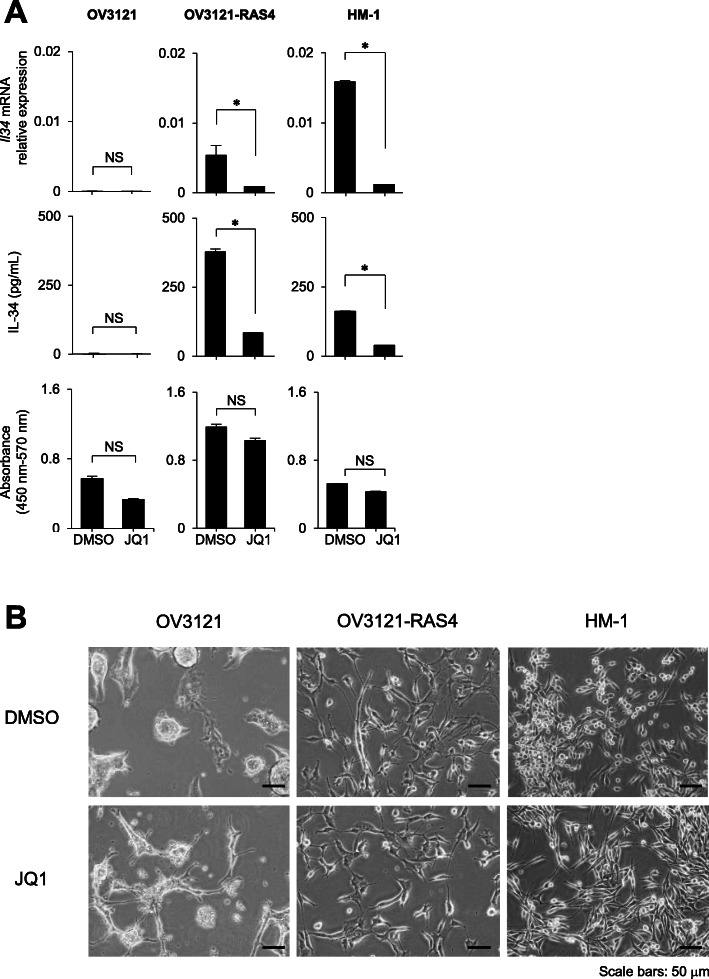
BET inhibitor JQ1 suppresses IL-34 expression in IL-34-expressing cancer cell lines. **a** Analysis of *Il34* mRNA expression (normalized by *Actb*, top panel), IL-34 protein concentration in the culture supernatant (middle panel), and cell viability (bottom panel) of mouse ovarian cancer cell lines OV3121, OV3121-RAS4, and HM-1 treated with JQ1 or DMSO (*n* = 3). NS, not significant; *p < 0.05; Student’s *t* test. Error bars represent SEM. **b** Morphologies of the cells treated with DMSO or JQ1. Scale bars 50 μm

We then tested whether JQ1 could really inhibit IL-34 expression in cancer cells. Murine ovarian cancer cell lines, OV3121, OV3121-RAS4, and HM-1, were treated with 10 μM of JQ1 for 2 days. OV3121-RAS4 and HM-1 spontaneously expressed IL-34 while OV3121 did not. Quantitative PCR and ELISA were performed to analyze mRNA and protein expression, respectively. As expected, when treated with JQ1, mRNA as well as protein expression of IL-34 was strongly inhibited in OV3121-RAS4 and HM-1 cells **(**Fig. 1a), whereas no change was observed in OV3121 cells (Fig. 1a). MTT assay was performed to ensure that IL-34 reduction was not due to decrease of cell viability (Fig. 1a). Cells were observed microscopically, and there were no obvious morphological changes according to the treatment with JQ1 (Fig. 1b). These findings indicate that, for the first time, IL-34 expression can be downregulated with a BET inhibitor JQ1 at both mRNA and protein levels.

### JQ1 suppresses Brd4 occupancy at Il34 gene promoter region

**Fig. 2:**
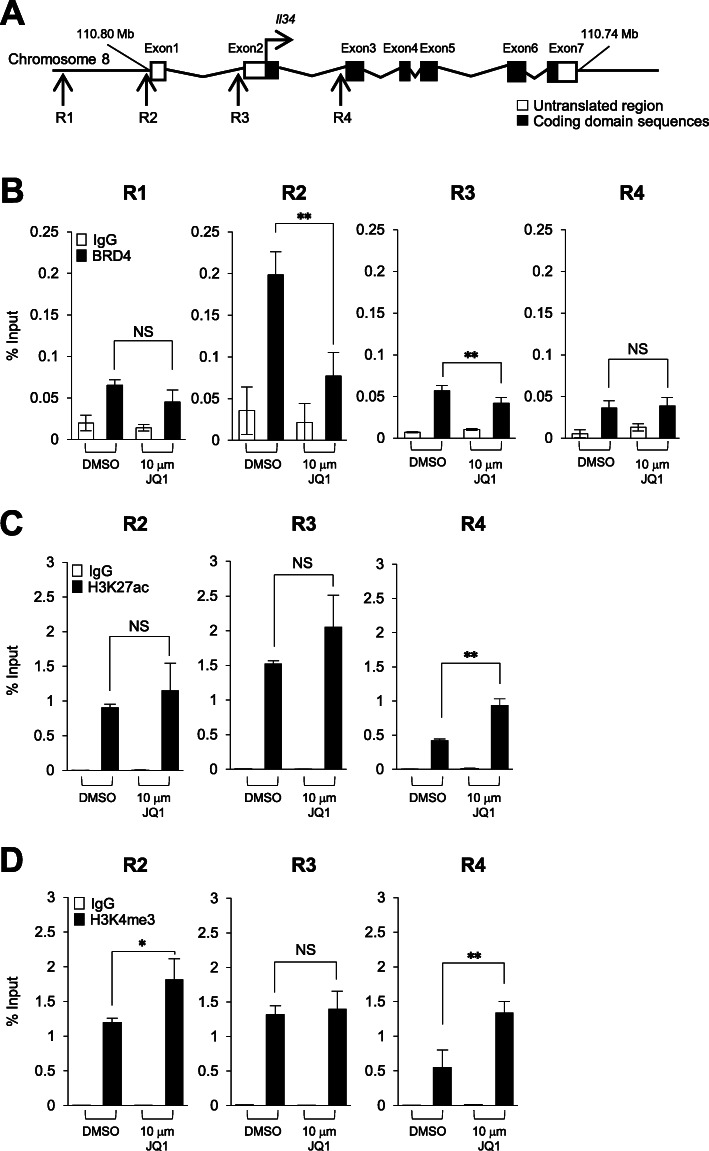
JQ1 decreases Brd4 occupancy at the *Il34* gene promoter region**. a** Schematic representation of mouse *Il34* gene locus. White and black boxes indicate untranslated region and coding domain sequence of *Il34*, respectively. R1 to 4 are possible promoter regions according to the Ensembl Genomes database. **b** Percent of input values of Brd4 in HM-1 cells analyzed by ChIP-qPCR. Genomic DNA from 10 μM of JQ1 or DMSO treated HM-1 cells was immunoprecipitated with anti-Brd4 antibody or control IgG and amplified by qPCR with *Il34* each region’s primer pairs. Representative results as mean ± SEM (*n* = 3) from two independent expreriments. NS, not significant; **p < 0.01; Student’s *t* test. **c** Percent of input values of H3K27ac in HM-1 cells analyzed by ChIP-qPCR. Genomic DNA from 10 μM of JQ1 or DMSO treated HM-1 cells was immunoprecipitated with anti-H3K27ac antibody or control IgG and amplified by qPCR with *Il34* each region’s primer pairs. Representative results as mean ± SEM (*n* = 3) from two independent expreriments. NS, not significant; **p < 0.01; Student’s *t* test. **d** Percent of input values of H3K4me3 in HM-1 cells analyzed by ChIP-qPCR. Genomic DNA from 10 μM of JQ1 or DMSO treated HM-1 cells was immunoprecipitated with anti-H3K4me3 antibody or control IgG and amplified by qPCR with *Il34* each region’s primer pairs. Representative results as mean ± SEM (*n* = 3) from two independent expreriments. NS, not significant; *p < 0.05, **p < 0.01; Student’s *t* test

It has been shown that JQ1 binds to BRD4 with high affinity and inhibits its binding to RNA POL II in malignant cells [20, 30]. Thus, it is required to confirm whether BRD4 can be recruited to the *IL34* promoter region. We carried out chromatin immunoprecipitation (ChIP) to confirm the involvement of Brd4 in the regulation of the *Il34* gene. According to the Ensembl Genomes database [31], the chromosomal location of mouse *Il34* is from 110,741,829 to 110,805,949 on chromosome 8. Untranslated regions are located in exon 1, 2, and 7, and coding domain sequences are located in exon 2 to 7. We determined four possible regions for the *Il34* promoter (R1 to R4) (Fig. 2a). ChIP analysis indicated that Brd4 occupancy was enriched at the R2 region that was significantly suppressed by JQ1 treatment (Fig. 2b). These data indicate that Brd4 is directly involved in *Il34* gene transcription and regulates *Il34* expression mainly by binding to the R2 region.

As Brd4 is known to bind acetylated lysine of H3 [20], we analyzed histone acetylation on several regions of the *Il34* gene including the R2 region which was suggested as promoter region (Fig. 2b) and the other regions (R3 and R4). Histone 3 lysine 27 acetylation (H3K27ac) modification was detected in all regions, and JQ1 treatment tended to slightly increase rather than decrease it (Fig. 2c). Further, histone H3 trimethylated at lysine 4 (H3K4me3) modification was analyzed. Similar to the results of H3K27ac modification analysis, H3K4me3 modification was detected at all regions tested, and JQ1 treatment tended to slightly increase rather than decrease it (Fig. 2d).

Collectively, our results suggest that regulation of *Il34* expression by JQ1 is mainly exerted by inhibition of Brd4 binding to the promoter region rather than by histone modification.

### JQ1 treatment suppresses Il34 expression in vivo and impedes tumor growth

JQ1 has been reported to inhibit growth of several types of tumors in vivo [25, 26]. However, it has not been known whether the anti-tumor effect of JQ1 was mediated by IL-34 suppression. In our previous research, we have shown that blockade of IL-34 could significantly suppress the growth of IL-34-producing tumors [8]. Therefore, we next sought to test the effect of JQ1 on tumor growth in vivo.

**Fig. 3:**
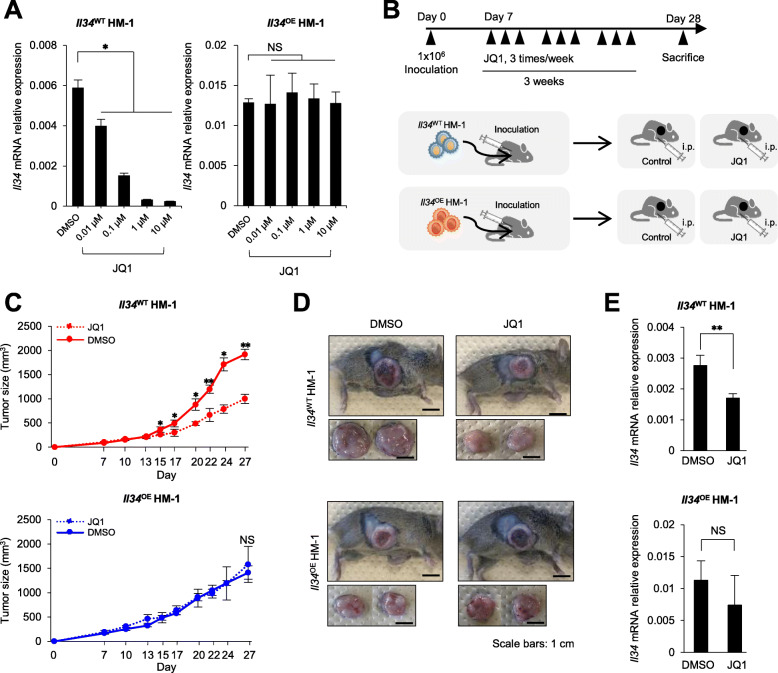
The anti-tumor effect of JQ1 is at least partly mediated by IL-34 suppression. **a***Il34* mRNA expression in *Il34*^WT^ HM-1 or *Il34*^OE^ HM-1 treated with JQ1 (*n* = 3). Data represent mean ± SEM. NS, not significant; *p < 0.05; Student’s *t* test. **b** Experimental design of JQ1 treatment in vivo. Mice were inoculated with wild-type HM-1 cell line which spontaneously produces IL-34 (*Il34*^WT^ HM-1) and with *Il34*-overexpressing cell line (*Il34*^OE^ HM-1). Mice were treated with JQ1 or vehicle control for 3 weeks. **c** Tumor growth in B6C3F1 mice inoculated with *Il34*^WT^ HM-1 or *Il34*^OE^ HM-1 and treated with JQ1 or vehicle control. (control: *n* = 4, JQ1: *n* = 3). NS, not significant; *p < 0.05, **p < 0.01; Student’s *t* test, error bars represent SEM. **d** Macroscopic observation of subcutaneously injected tumors in B6C3F1 mice sacrificed on day 27. Scale bars 1 cm. **e** The expression level of *Il34* normalized by *Gapdh* in HM-1 tumors collected on day 27 (control: *n* = 4, JQ1: *n* = 3). NS, not significant; *p < 0.05, **p < 0.01; Student’s *t* test. Error bars represent SEM

HM-1 is known as aggressive and highly metastatic tumor cells with poor prognosis [32]. As shown above, HM-1 cells spontaneously produce IL-34 (Fig. 1a). We designated an intact HM-1 cell line as *Il34* wild-type line (*Il34*^WT^). And we generated *Il34*-overexpressing cell line (*Il34*^OE^) driven by *EF1α* promotor whose activity is not inhibited by BET inhibitors [33]. These cell lines were treated with JQ1 in vitro*,* and the *Il34* expression was efficiently suppressed in a dose-dependent manner only in *Il34*^WT^ but not in *Il34*^OE^ (Fig. 3a). Additionally, we carried out ChIP analysis for H3K27ac to demonstrate histone acetylation in the introduced *EF1α* promoter. H3K27ac modification was detected in the introduced *EF1α promoter* in *Il34*^OE^, and JQ1 treatment did not affect it (Supplementary figure 2). These results indicate that only endogenous *Il34* expression was regulated by Brd4.

Then, tumor cells were inoculated into syngeneic B6C3F1 mice 1 week before the onset of treatment, then tumor-bearing mice were randomly divided into two groups (control or 50 mg/kg of JQ1, 3 times a week) (Fig. 3b). Tumor size was measured at the indicated time points during treatment (Fig. 3b). Notably, there was no significant difference in the tumor growth of *Il34*^OE^ HM-1 tumors regardless of JQ1 treatment. In contrast, JQ1 treatment significantly suppressed the tumor growth in the *Il34*^WT^ HM-1 group (Fig. 3c). We collected the tumor tissues at the endpoint of observation to analyze *Il34* mRNA expression. Consistent with the in vitro findings, *Il34* expression in *Il34*^WT^, but not in *Il34*^OE^, HM-1 tumor was remarkably reduced by the JQ1 treatment (Fig. 3e). Based on these results, it is strongly suggested that in vivo treatment with BET inhibitor JQ1 suppressed IL-34 expression in TME which leads to the anti-tumor effect.

## Discussion

IL-34 is known to affect proliferation and induction of therapeutic resistance in various types of tumors [5, 7, 8, 11]. Consequently, the regulation of IL-34 expression could be considered as a new therapeutic method. However, the mechanism of IL-34 expression in cancer cells has not yet been clarified. It has been indicated that IL-34 can be induced by various stimuli such as chemotherapy, chemical stressors, pro-inflammatory cytokines, PAMPs, vitamin D, and viral infections in a wide range of cells via an NF-κB-mediated mechanism. On the other hand, IL-34 expression is suggested to be downregulated by other stimuli such as transforming growth factor-beta 1 and bone morphogenetic protein 2 [14]. More recently, IL-34 has been suggested to be highly expressed in human mesangial cells of lupus nephritis patients and is negatively regulated by the Wnt pathway [34]. However, these reports are limited to describe which stimulations or pathways relate to the expression of IL-34, and IL-34 gene transcription regulator has been unknown.

In this study, we identified for the first time a responsible transcription regulator that controls *Il34* expression. We found that the BET inhibitor JQ1 efficiently suppressed *Il34* expression in cancer cell lines (Figs. 1a and 3a). JQ1 binds with a high affinity to the first bromodomain of BRD4 [30], and our results using mouse cell lines indicate that Brd4 is recruited to the promoter region of the *Il34* gene and controls its expression.

Although JQ1 treatment in HM-1 cells clearly reduced Brd4 binding to the promoter region and *Il34* expression, H3K27ac and H3K4me3 status in the region were not significantly changed (Fig. 2). These results indicate that JQ1 exhibited its suppressive function on *Il34* expression through inhibiting direct binding but not changing (decreasing) accessibility of Brd4 to the *Il34* promoter. Brd4 is known to recruit transcription elongation factor P-TEFb and facilitates transcription elongation [20]. In *Il34* gene expression, it is likely that the transcript elongation by Brd4 is impaired by JQ1 treatment.

It has been reported that Brd4 also affect enhancers [23, 35] or super-enhancers [35, 36]. In *Il34* gene, several enhancer regions are suggested according to Ensembl database [37, 38]; therefore, it is possible that JQ1 exerts its function as a therapeutic drug by regulating them in *Il34* gene. Regarding the super-enhancers, according to SE analysis [39], there are seven super-enhancer regions in the *IL34* gene in human cells, and BRD4 binding was observed in at least two regions. In mouse case, ten regions of *Il34* super-enhancers have been speculated in a database established by Khan and Zhang [40]. Therefore, JQ1 may also inhibit *Il34* super-enhancers, in addition to its promoters, and contribute to the therapeutic outcome by regulating *Il34* expression.

There are three future tasks to understand the detailed mechanism of IL-34 expression. Firstly, investigation of condition which promotes the binding of Brd4 to *Il34* promoter region is needed because the promoting process and factors are still unclear. As mentioned above, some reports suggested several pathways and transcription factors such as RUNX1 or NF-κB are important. Therefore, protein phosphorylation analysis of related factors could explain a process of IL-34 expression. Secondly, the data that BET inhibition did not completely suppress IL-34 expression (Figs. 1a and 3a) implies the existence of other pathways regulating IL-34 expression. Finally, although suppression effect of in vivo tumor growth by Brd4-specific inhibitor JQ1 was shown in Fig. 3c, it is necessary to investigate whether the growth suppression is a solely IL-34-dependent phenomenon because Brd4 regulates a wide range of tumor-associated transcription factors.

In conclusion, we identified here Brd4 as a responsible transcription regulator that controls *Il34* expression in cancer cells. The findings may help establish a new therapeutic strategy against IL-34-producing cancer in which usually an immunosuppressive environment is observed. Future studies on primary cancer tissues and cells are needed to strengthen this conclusion.

## Conclusion

According to the results, *Il34* expression mechanism is suggested to be regulated by Brd4 in IL-34-producing cancer cells, which is efficiently suppressed with JQ1. Treatment of mice bearing IL-34-producing tumors with JQ1 shows anti-tumor effect.

## Supplementary information

**Additional file 1: Supplementary Figure 1. **Effects of various inhibitors upon *IL34* mRNA expression in cancer cells. Normalized value of *IL34* expression in Vemurafenib (BRAF inhibitor)-treated melanoma cell line A375, SB216763 (Glycogen synthase inhibitor)-treated leukemia cell line RS4.11, Bortezomib (proteasome inhibitor)-treated breast cancer cell line MCF7, Imatinib (tyrosine kinase inhibitor)-treated leukemia cell line EOL-1, SAHA (histone deacetylase inhibitor)-treated ovarian cancer cell line SKOV-3, JQ1 (BET inhibitor)-treated MYCN-amplified neuroblastoma cell line CHP-212, and PD-0325901 (MEK inhibitor)-treated malignant peripheral nerve sheath tumor tissue from the NCBI database.

**Additional file 2: Supplementary Figure 2. **Analysis of histone acetylation in *Il34*^OE^ HM-1 derived by *EF1α* promoter. Percent of input values of H3K27ac in *Il34*^OE^ HM-1 cells analyzed by ChIP-qPCR. Genomic DNA from JQ1 or DMSO treated HM-1 cells was immunoprecipitated with anti-H3K27ac antibody or control IgG, and amplified by qPCR with *EF1α* promoter region’s primer pairs. Data represents as mean ± SEM (n = 3). ND; not detected. NS; not significant, Student’s t-test.

## Data Availability

All data generated or analyzed during this study are included in this published article.

## Abbreviations

IL: Interleukin
BRD: Bromodomain-containing protein
TME: Tumor microenvironment
CSF-1: Colony-stimulating factor-1
CSF-1R: Colony-stimulating factor-1 receptor
RUNX1: Runt-related transcription factor1
CDK: Cyclin-dependent kinase
BET: Bromodomain and extra terminal domain
Pol II: RNA polymerase II
P-TEFb: Positive transcription elongation factor b
PAMPs: Pathogen-associated molecular pattern
ChIP: Chromatin immunoprecipitation
