# Supplementary figures and images for: Bromodomain-containing protein 4 regulates interleukin-34 expression in mouse ovarian cancer cells
Source: Inflamm Regen. 2020 Oct 14;40:25. doi: 10.1186/s41232-020-00129-4 (PMC7556959; doi:10.1186/s41232-020-00129-4)

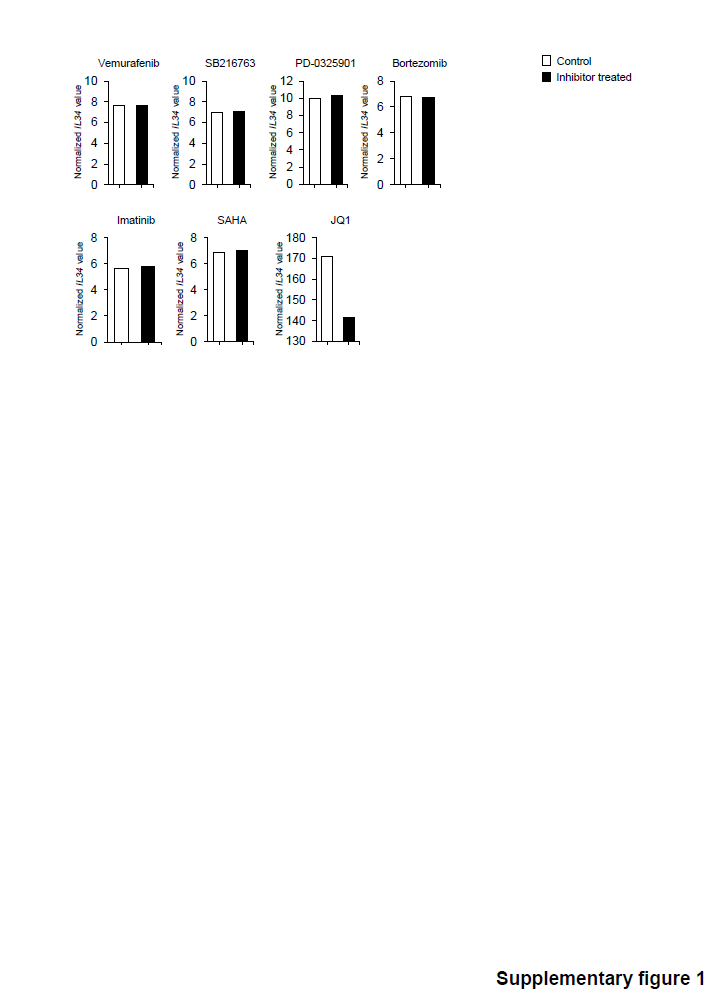

Supplement: Supplementary file 1 — Additional file 1: Supplementary Figure 1. Effects of various inhibitors upon IL34 mRNA expression in cancer cells. Normalized value of IL34 expression in Vemurafenib (BRAF inhibitor)-treated melanoma cell line A375, SB216763 (Glycogen synthase inhibitor)-treated leukemia cell line RS4.11, Bortezomib (proteasome inhibitor)-treated breast cancer cell line MCF7, Imatinib (tyrosine kinase inhibitor)-treated leukemia cell line EOL-1, SAHA (histone deacetylase inhibitor)-treated ovarian cancer cell line SKOV-3, JQ1 (BET inhibitor)-treated MYCN-amplified neuroblastoma cell line CHP-212, and PD-0325901 (MEK inhibitor)-treated malignant peripheral nerve sheath tumor tissue from the NCBI database. [file 41232_2020_129_MOESM1_ESM.docx]

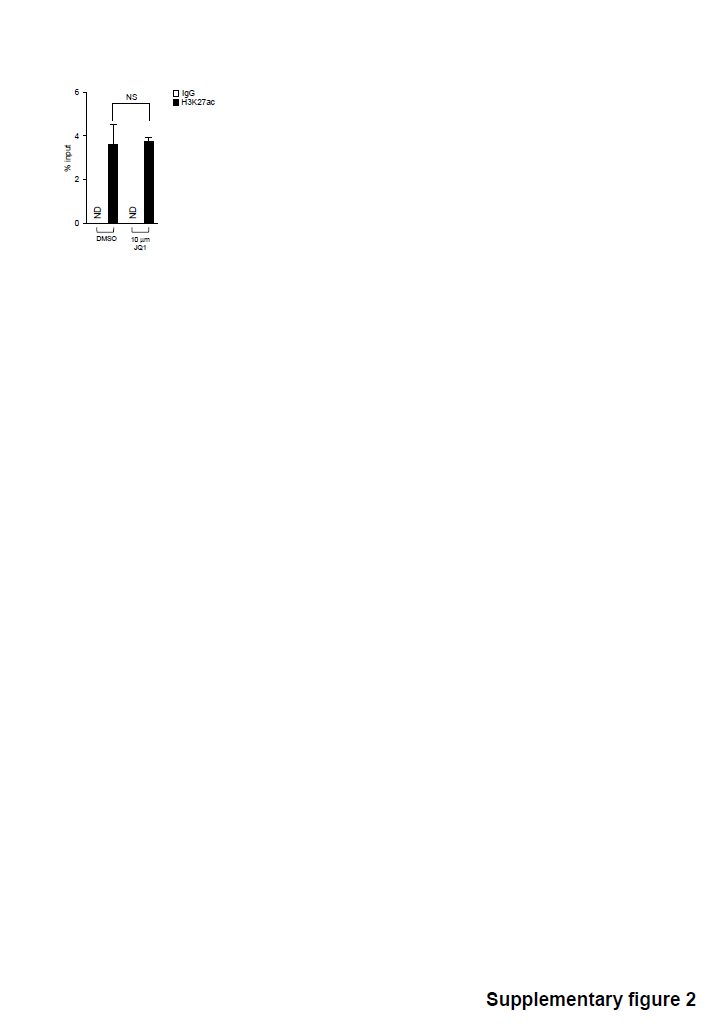

Supplement: Supplementary file 2 — Additional file 2: Supplementary Figure 2. Analysis of histone acetylation in Il34OE HM-1 derived by EF1α promoter. Percent of input values of H3K27ac in Il34OE HM-1 cells analyzed by ChIP-qPCR. Genomic DNA from JQ1 or DMSO treated HM-1 cells was immunoprecipitated with anti-H3K27ac antibody or control IgG, and amplified by qPCR with EF1α promoter region’s primer pairs. Data represents as mean ± SEM (n = 3). ND; not detected. NS; not significant, Student’s t-test. [file 41232_2020_129_MOESM2_ESM.docx]
